# On the Conveyance of Cholera from India through Persia, Armenia, and Georgia, to Russia and the Rest of Europe

**Published:** 1868-09

**Authors:** John C. Peters

**Affiliations:** New York


### CHICAGO MEDICAL EXAMINER.

N. S. DAVIS, M.D., Editor.

VOL. IX. SEPTEMBER, 1868. NO. 9.

### Original Contributions.

ARTICLE XXX.

### ON THE CONVEYANCE OF CHOLERA FROM INDIA THROUGH PERSIA, ARMENIA, AND GEORGIA, TO RUSSIA AND THE REST OF EUROPE.

By JOHN C. PETERS, M.D., of New York.

There is a great nursery of cholera in the northern part of Hindostan, at the foot of the Himalaya Mountains, *viz.*, at Hurdwar, where the thrice sacred Ganges emerges from the mountains, and from being a tiny stream becomes a river, and descends upon the land which it is to fertilize and bless. The largest of all the Hindoo festivals and fairs is held at Hurdwar every year, at the time of the vernal equinox, *viz.*, in April, when at least 200,000 or 300,000 pilgrims and merchants always assemble there. But every twelfth year, which is particularly holy, as many as 1,500,000 to 3,000,000 of devotees and traders are often crowded together. This festival is not only the focus for the produce of all Hindostan, from Calcutta in the south-east, and Bombay in the south-west, and all the intermediate cities and provinces; but merchants and pilgrims also hasten there from Arabia, Persia, Beloochistan, Afghanistan, Cabul, Independent Tartary, Central Asia, and Russia.

As a matter of course, cholera is often brought to Hurdwar; often originates there; and is frequently carried away from it, not only down the Ganges, Jumna, and Indus rivers, but also up to the north and west, *viz.*, to Afghanistan and Cabul, and from thence to Persia and Independent Tartary and Russia. It leaves India in three directions. The principal line of travel is from Hurdwar to Lahore, from there to Attock and Peshawur, through the great Kyber Pass to the city of Cabul. The second is from Hurdwar to the city of MOULTAN, through the Bolan Pass to Kelat in Belochistan, and to Candahar in Afghanistan. A third stream of the disease is carried down the river Indus to Kurrachee and Bombay, and from there forwarded over to Arabia, and up the Persian Gulf and Red Sea.

In 1783, over 1,500,000 devotees assembled at Hurdwar, and in eight days over 20,000 of them died of cholera. Twelve years after, *viz.*, in 1795, the same thing occurred in a lesser degree; also in 1807. In 1867, *viz.*, 5 times 12, or 60 years afterwards, the *London Lancet* tells us 3,000,000 of Hindoos assembled at Hurdwar, and, in consequence, a very disastrous outbreak of cholera took place.

The cholera of 1827 destroyed 30,000 persons in Lahore, then passed north through Attock and Peshawur and reached Cabul, from whence it was distributed due west to Herat, Meschid, and Teheran, and reached Russia, at Astrakhan, in 1829 and 1830. From Cabul it also passed north, to Balk, Bokhara, and Khiva, and reached Orenburg, in Russia, in 1829.

The epidemic of 1841 began its second great emigration, after prevailing in a frightful manner along the rivers Ganges and Indus, reached Lahore, was carried to Cabul, and conveyed, by pilgrims, from Herat, in November, 1845, to the holy city of Meschid; from there it went to Teheran, in June, 1846, and prevailed so frightfully as to kill 9000 persons in four months. From Teheran it was again carried to Russia, in 1846 and 1847. From Cabul it was also carried to Balk, Bokhara, and Samarcand in 1845; and to Khiva and Orenburg in 1846.—DRASCHE.

The great pandemic of 1849 passed along the same routes, killing 15,000 persons in Teheran in 1851, and 11,000 more in 1852 and 1853. We have given these dates and figures solely to prove the importance of attention to these great routes of cholera towards Europe from the north-west provinces of Hindostan.

CABUL is the first great town to the north-west over the borders of the British possessions in India. It is the grand centre of the trade of India with Persia, Central Asia, and Russia. It is the southern terminus of the Russian commerce with Hindostan, and has long been the theatre of the commercial and political rivalry of Russia and England, in their attempts to control Persia and Central Asia. Cholera is regularly brought up to Cabul from Hurdwar, through Lahore, Attock, and Peshawur, and, although it is called "the city of 100,000 gardens," parts of it are well adapted to multiply the infection of cholera. Many of its small streets are built over, forming low, dark tunnels, containing every offensive thing. Water collects and stagnates in ponds all over the city; the inhabitants cast out the refuse of their houses into the streets; and dead dogs and cats are frequently seen lying on heaps of the vilest filth, while dead horses occasionally pollute the air for many days together. —Sir ARTHUR CONNOLLY.

The traffic between Russia, Persia, Central Asia, and India, by way of Cabul, is made under curious conditions, which have descended from high antiquity; for it belongs to four tribes of Lohanee Afghans, called *Provindahs*, who are both pastoral and mercantile in their pursuits, and number 8000 families, with over 30,000 camels and 10,000 oxen of transport. They organize themselves into three immense caravans, which resemble veritable *corps d'armee*, and descend into India, not only in time to attend the great fair at Hurdwar, but also to penetrate down the Ganges to Benares, and along the Indus to Kurrachee, and thence by sea to Bombay. They bring down Russian, Persian, and Afghanistan articles of trade, and return with cotton goods, muslins, shawls, silks, brocades, and innumerable other articles. The first great division has 20,000 to 30,000 camels, numerous oxen, and 50,000 to 60,000 head of sheep; the second division, 10,000 to 17,000 camels and a proportionate number of oxen and sheep; and the third division, 3000 to

5000 camels. These all return to Cabul and Candahar by the middle of June, in time to dispatch their investments on to Herat and Meschid in the west, and to Balk and Bokhara in the north-west. They also always descend again into India by the end of October, with 36 different Russian articles, and return with 64 kinds of English and India goods. From 1500 to 2000 camels are alone employed in conveying pomegranate rinds, which dress leather in a superior manner; 3000 camels carry coarse cotton cloths from India to Cabul; five caravans with shawls, from the 16,000 looms in Cashmere, have arrived in Cabul in one year; much salt is carried up from the inexhaustible mines at Lahore; many thousand camel-loads of fresh and dried fruits are brought down to India; and Russian goods are so cheap and common in Cabul, at times, that looking-glasses, large enough to serve as window-panes, are sold for half a dollar. In addition, there is a great Hindoo festival on the banks of the Indus, near Attock, every April, to which great crowds congregate. Pilgrims also come down from Cabul to the river Indus, which they descend to the sea and then pass on to Mecca; and a great Mohammedan festival takes place on the 28th of March. Such are the causes which propel cholera up from India to Candahar and Cabul.—Sir ALEX. BURNES.

From Cabul, the disease is always carried due west to HERAT. Occasionally, it has happened that a single traveller or pilgrim could proceed, unarmed, from Cabul to Herat. But, generally, the merchants and devotees travel in great caravans, with long strings of camels. They are armed to the teeth, and often bear the marks of many a conflict. Now and then some prince, like the son of the King of Lucknow, comes up with a large suite of Indo-Mohammedans; and it is thought that over 60,000 pilgrims pass through Herat every year, on their way west to the holy city of Meschid.

Herat has at least 50,000 inhabitants, and is a well-known town; but in 1857, the *London Times* asked, "Where is Herat?" It is in the extreme north-western corner of Afghanistan, and stands in the same relation to this province that Peshawur does to India; for it is the only gate by which it can be approached from the west. All the practicable roads from Persia and Central Asia converge and unite as they approach Herat. It is the door and citadel which must be opened before caravans and armies from Persia and the west can reach Afghanistan and Hindostan. Its situation is one of the greatest military and commercial importance; for the peaceful files of the caravan and the dread battalions of war must alike pass through it on their way from India to Persia, and from Persia to India. Long camel trains, coming up from Delhi, Moultan, Hurdwar, and Lahore, traverse it, bearing the products of India and the manufactures of England to the distant towns and oases of Persia and Turkistan. So completely is Herat a gateway of commerce, that it is called a "*bunder*," or port, although the only seas upon which it borders are seas of sand. The march of conquest has led through it from time immemorial, especially after the time of Mohammed. Since A.D. 710, host after host of fierce warriors, led by the Kaliphs of Damascus and Bagdad, of Syria and Western Persia, coming almost from the borders of the Caspian, Black, and Mediterranean Seas, have carried the tassels of the Mohammedan flag victoriously down into India. The vast and fertile plain about Herat has often been a huge *place d'armes*, where all the assembled columns from the west have united and recruited before making their final descent upon Hindostan, and it has often been predicted that the Cossack and the Sepoy must, sooner or later, meet there in deadly strife. No better camping-ground and quarters have ever been found for caravans and armies. Abundant crops of wheat and barley, and every kind of fruit known to Persia, grow there in profusion. Cattle and sheep abound. The bright waters of the river are as "clear as tears," and numerous running streams and artificial canals light up the pleasant landscape and fertilize the soil. Such is the profusion of roses, that it is called the "*City of Roses*," and a very large proportion of the whole world's supply of the celebrated "Attar" comes from it. But there are other smells than those from roses about Herat. At night, on the road, one is apt to be wakened by the shouts of drivers and the tinkling of bells, announcing a passing caravan, and by the faint light of the moon perceives a sea of long, black boxes surging by on scores of mules and camels. Each animal is laden with two of these horrible objects, one on each side, and many of them are so loosely nailed together that another sense than that of smell soon convinces one that they are used as coffins. In fact, they contain the bodies of the devout, who, having died in the true faith, are now being taken to be buried in holy ground at Kerbela or Meschid. They are often carried hundreds of miles, and a sickening stench always comes up from their gaping fissures, causing nausea and faintness in the drowsy, unsuspecting traveler, who finds it impossible to extricate himself promptly from their neighborhood; for they come crowding on in the dark as if there were no limit to their numbers.

The cholera of 1828 swept away many thousands in Herat, although water, that prime necessity of Oriental life, is so abundant that almost every house has its fountain, besides the large public ones in the streets and bazaars. Each successive epidemic of the disease in Hindostan has reached Herat, and been carried on through Persia to Turkey and Russia. That of 1841, reached Cabul in June; Herat in August; Meschid in September; and Teheran in October.

The next important town due west of Herat is MESCHID. This is so holy that no person of any sect called Mohammedan has ever dared to commit the impiety of firing a hostile shot at its walls. For eight months in the year all the roads to and from Meschid are thronged with pilgrims. Nearly 60,000 come up from India, Cabul, and Afghanistan; and as many more from the south of Persia. Over 100,000 crowd on fanatically from Turkey in Asia and the Caucasus in the west; and perhaps an equal number from the north.

Connolly says, the distant cities in which the pilgrims assemble and make themselves up into caravans, to go to Meschid, are so thronged with men and animals, that there is scarcely passage through the crowd. Horses, mules, camels, and cattle are picketed the entire length of the outer walls, where they neigh, bray, and fight, while their masters are busy currying or shoeing them; mending pack-saddles; higgling for supplies of straw or corn; or sleeping, praying, or reading the Koran.

The pilgrims generally travel at night; some of them carry torches, and at the head of the line a pot of live charcoal swings under the belly of a horse, to light their pipes and mark the line of march. In cool and pleasant weather they move by day. Gay pennons are then unfurled; and all go on with light hearts. From time to time, their leaders raise a shout for the blessed Mohammed, and if their followers do not join in loudly and unanimously, they are urged to do "better than that," "sweeter than that," angels are called upon to aid them, till, finally, the air is rent with fervent and inspiring cries, which makes one's very heart's blood boil. Going and returning pilgrims greet each other on the road. Many others are seen encamped at the road-side; the poorer pilgrims in miserable tents and shelters, but the richer Turks and Persians in green, sky-blue, or white tents, surmounted by gilt balls and crescents, and lined with costly carpets. Well-clad male and female servants, in numberless groups, walk, recline, sit, smoke, or chat in and about the camps. Hundreds of horses, richly caparisoned, graze around. Huge piles of baggage and provisions are guarded by trusty soldiers, bristling to their chins with silver-hilted daggers and inlaid pistols. The whole bearing an air of wealth, pomp, and security.

The scene is more wild and strange when the pilgrims halt at caravansaries. The noise and quarreling, at first, can only be imagined by those who have seen Persians on the march. But the leader of the caravan soon takes his place in the middle of the square, and commences reciting prayers and verses in honor of the Prophet, to which the pilgrims shout short sentences from the Koran in reply. Connolly says, the effect of their voices coming from the cells on all sides is very wild and pleasing, especially at night, when the sounds gradually rise, chiming in with each other till they are perfectly blended in one full chorus. But soon all fall asleep and silence reigns, in cells which are so filthy that few Europeans can remain in them.

Finally, the holy city is reached, and several times in the course of every day dense troops of soiled and jaded pilgrims and travelers pass through the city gates of Meschid, into the great square, which is usually crowded with people from all parts of the East: with Afghans, Arabs, Koords, Turks, Osbegs; with pilgrims from all the provinces of Persia; with priests, merchants, peasants, and dervishes, without number, fromt he borders of the Caspian and Black Seas, and from the Persian Gulf. The great and magnificent square is approached from two sides by very high arched gates, of exquisite architecture, faced with brilliant blue-enameled tiles. On the other two sides are deep arched porches, of the same height and proportions as the gates, but covered with copper tiles, heavily gilt. One porch leads into a fine mosque; the other faces a high, gilded minaret, and the golden dome under which rest the sacred remains of the Iman. The space between the gates and porches is enclosed on all sides by a double story of arched cloisters, fronted with mosaic work and paved with the gorgeous gravestones of rich and holy men. A stone canal conducts water through the centre of the square, for the ablutions of the faithful, and beautiful shade trees abound. The midday sunbeams are reflected gorgeously from the many-colored and golden tiles; and the light of the declining luminary falls glowing upon the resplendent dome. But the most glorious effect is produced when rain falls while the sun is breaking through light clouds, causing the large drops which are shed from the golden tiles to glitter like enormous diamonds. This sparkling shower on the gilt dome is often worshipped as a down-pour of heavenly light, and excites the pilgrims to a perfect frenzy of enthusiasm.

But Meschid presents other sights and sounds than those of religion and pleasantness. Hundreds and thousands of beggars, of the most miserably squalid appearance, beset every approach to the shrine, waylaying the pilgrims in every direction. Old men and women, in the most abject states of want and misery, throng the streets, which are also strewn with children, not more than two or three years old, grovelling in the dirt, mere living skeletons, more like starved young animals than human creatures. Some are crying and sending forth piteous petitions with their little half-quenched voices; others, silent, lying like dead things; others, listless and motionless, but with a wolf-like glare of hunger in their sunken eyes, giving terrible evidence of the fierce pangs which gnaw within them. Blear-eyed girls and filthy boys carried things like starved cats in their arms, all squalling for bread. Fraser ran the gauntlet of a crowd of spectres of this kind, nearly half a mile long. Most of them were the wives and children of Turcomans from the eastern borders of the Caspian Sea, whose towns had been sacked by the Meschidees. The able-bodied men and women had been sold as slaves, and the old people and children thus left to beg or starve. As many as 3000 to 4000 persons have been brought into Meschid at one time, and the feeble left to the tender mercies of pilgrims and strangers.

Cholera is always carried from Meschid to Astrabad, on the east coast of the Caspian Sea, by merchants and pilgrims, and then forwarded up to Astrakan. It is also conveyed from Meschid to Teheran, the capital of Persia, situated only 70 miles south of the lower border of the Caspian Sea.

TEHERAN is one of the most important centres for the reception and distribution of cholera. The disease not only reaches it from Meschid and the east, but also comes up from the Persian Gulf, by way of Bushire, Shiraz, and Ispahan. Although Teheran has 80,000 inhabitants, no city in Persia makes so poor an appearance on first approach. There is not a dome or minaret of any size to decorate it; nor is it surrounded by woods and gardens, like Damascus. All that meets the eye is a line of mud walls and bastions, surrounded by many ruins, in the midst of a gravelly plain bounded by rugged mountains. The streets are narrow, unpaved, and exceedingly filthy. The bazaars are extensive, but wretchedly kept and very dirty; and the climate is so unhealthy in the summer, that the greater part of the inhabitants are obliged to leave the city.

From Teheran, cholera is always carried due north to RESHDT, at the foot of the Caspian Sea, whence it is distributed to every port on the west coast of the Caspian, *viz.*, Leucoran,

Saljan, Baku, Derbent, Kisliar, and Astrakan. It also passes up all the rivers which empty into the Caspian, and thus invades the whole country between the Caspian and Black Seas.

From Teheran, the disease is always carried north-west, to Kasbin and the great city of Tabriz.

TABRIZ has 80,000 inhabitants, and is the most important city, in a commercial point of view, in all Persia, for it is the great mart of European merchandise. The transit trade to and from Persia is immense, almost beyond calculation; and it is the grand depot into which Russia, Germany, Turkey, and England pour the fruits of their industry and enterprise, to be distributed to the vast regions beyond. Its bazaars and caravansaries are numerous and extensive, built of brick and lime, finely arched, and among the most durable structures in the world. But its streets are narrow, crooked, irregular, only partially paved, and very sombre, for no windows open from the houses towards the streets, which present nothing to the eye but a dull line of mud walls, eight to fifteen feet high, pierced by gates and doors so low that one must stoop to enter them. But the houses of the rich are beautifully finished inside, and have fountains in their beautiful court-yards surrounded by trees and shrubs and filled with singing birds. Even the poorer houses have open rills and running streams in their yards, conducted there from the larger canals. The climate is delightfully healthy; warm by day, but deliciously cool at night, when a strong breeze blows diurnally from the Caspian. It is elevated 4000 feet above the level of the sea, and looks down from the head of a vast amphitheatre upon a fertile plain, 30 miles wide. Scarcely a cloud appears in the clear atmosphere from early spring till late in the autumn, and the whole neighborhood is so healthy that the name of Tabriz is derived from *Tab* (fever) and *reektan* (dispersing); yet, cholera is always conveyed to it from Teheran. It appeared there in 1823; again, in 1828 and 1829, and killed 5000 persons; in August and September, 1845, it is said to have killed 400 in one day; and every successive pandemic has reached it, by means of merchants, travelers, and pilgrims, going to and from Meschid and Mecca.

From Tabriz, cholera is always carried due north, through Erivan to TIFLIS, and thence up to Astrakan, on the Caspian; and also north-west, to Erzeroum, and from thence to TREBIZOND, on the Black Sea.

ERZEROUM, to the north-west of Tabriz, is one of the oldest cities in the world. It is situated at the base of the Ararat range of mountains, at the head-waters of the Euphrates, and claims to have been founded by a grandson of Noah. Its trade is immense, as it is situated felicitously for the transit of goods from Europe, Asia Minor, Syria, Persia, Turkey, and Russia. Almost weekly, a commercial caravan starts for Tabriz, consisting of from 300 to 900 horses, laden with English, German, and Russian goods, along a road laid out by the ancient Genoese, and still marked by the ruins of their forts.

A caravan of horses generally starts at 2 A.M. The guide, or Karavan Baschi, gives the signal by a peculiar cry, and the horses respond by a general snorting, neighing, and tinkling of the bells on their necks. Notwithstanding the thick darkness of the night, such is their intelligence that each horse soon finds his owner or driver, and stands motionless until the pack-saddles and bales of goods are duly balanced and fastened on its back; then starts off on his own accord, finds his proper place in the train, in files two abreast. The oldest and most experienced horse takes the lead, proud of the distinction, tossing his larger string of bells, of which every fifth or sixth horse has a smaller one, the tones of which are cheerful, if somewhat monotonous. Thus they wend their patient way, under their huge burdens, stretching in files of miles in length, over hills and through valleys, never straying or shying, and displaying an instinct, almost amounting to reason, in overcoming obstacles, and never attempting to cross a stream until the proper ford has been indicated to them. In winter they are obliged to push on from 20 to 25 miles a day, to reach shelter; but in summer, they find abundance of forage, and travel more leisurely. This may not be the sublimity of commerce, but it forms a pleasant scene. The muleteers are good Mohammedans, who never seek retirement for their prayers, but commence at the moment prescribed. Wherever they may be on the road, they tumble off their horses, face towards Mecca, commence their prayers, then rise and ride on quickly to overtake the train, give a few directions, mingled with more curses, to their underlings; dismount again to resume their devotions, repeating this process, interlarded with many blows upon their horses, until the customary prayer is all completed. Quip, crank, laugh, and roundelay pass along the line all the march, and at night, in summer, the merry muleteer sleeps upon the bare earth. In the winter, they crowd into huge sheds, the front part of which is used as a stable, the rear as a hotel. These dirty holes have platforms raised 2½ feet above the mud floor, to enable the guests to keep out of the litter and dung which carpet the floors of these elegant refectories. All the platforms are covered with mats, decayed through age and filth, and equally old and dirty mattresses and pillows. Upon these, the travelers sleep in their wet cloaks and dirty boots, forming a loathsome combination.

From Erzeroum, cholera is always carried to TREBIZOND by caravans, in which 26,000 horses were employed in 1834, some merchants using from 500 to 1000, each carrying 300 pounds. Several times a week one of these cavalcades arrives, pouring itself into some of the huge caravansaries, and depositing its heavy loads of Persian and other goods in huge piles, almost like small mountains. And quite as often, equally large trains set out for Erzeroum. The scenic effects of the town are very great in every direction, whether we look up at it from the sea, or down upon it from the lofty hills behind and observe the Black Sea, the city and surrounding country spread out in a picture of almost unequalled beauty and grandeur. The view extends along a broken line of coast, from whence rise lofty mountains, piled one upon another, until they reach the snow. Although the streets are exceedingly narrow, crooked, and filthy, like those of all Eastern towns, yet they have charms peculiar to Trebizond; for, in riding along, one often suddenly emerges upon one of many romantic bridges which span deep ravines, at the bottom of which tiny rivulets run down to the sea, and the precipitous sides of which are covered with dense foliage and vines which cling to lofty trees and clamber up to overrun the walls of castles perched upon their dizzy brinks.

From Trebizond, which is almost the only port at the south-east end of the Black Sea, cholera is regularly carried by sailing and steam vessels to Constantinople, Sebastopol, and Odessa, and also up the Sea of Azof to the mouth of the River Don.

To return to Tabriz: we have already seen that cholera is always carried up between the Black and Caspian Seas to Erivan and Tiflis; and from Tiflis to Astrakan on the Caspian, and the towns of Azof, Tagonrog, and Tcherhask on the Sea of Azof, and thence distributed up into Russia. This is now so well known that it requires no proof. But we must give a description of the *houses* in Persia, Armenia, Georgia, and Circassia, which will account for much of the terrible ravages which cholera always commits when it reaches these countries. The structures are still made and the people live exactly in the manner described by Xenophon, who traversed this country and embarked at Trebizond with the immortal 10,000. In the summer-time, in riding along, one often finds one's self on the top of hillocks covered with grass, upon which cattle are grazing; but smoke, issuing from piles of stones a few feet high, makes one suspect that he is riding on the tops of houses and over a village; and soon, from holes in the ground, like rabbits from their burrows, men, women, children, and dogs rush up in abundance. Then one descends into a yawning cavern and dark gulf, filled with smoke, dust, glare of torches, uproar of lowing and bleating beasts, crying children, crowing cocks, and gabbling women, until one believes that he has got into a modern Noah's ark anchored in the ground. The place is dark as pitch, even at noonday, and the bewildered stranger is led along, continually slipping in fresh cow-dung. These vaults sometimes cover an acre of ground, and are divided into an interminable number of rooms and cellars. A multitude of cows and calves often stream out unexpectedly from some side room, and nearly upset and suffocate one in the ocean of their filth. Sheep are herded in other nooks, and lambs trot all about and into the women's apartments. The horses are stabled more carefully, and a collection of unlovely women and children occupy separate apartments, into which only dirty men, a few lambs and calves, various chickens, or a favorite filly, occasionally intrude. Old men are found sitting about, with long white beards like those of the shepherds of Lot and Abraham. Fraser and Fisher, who often lodged in these places, were generally obliged to turn out some favorite horse or pet cow, in order to obtain sleeping room; the ground was then swept over, and dirty rugs were placed for them to sleep upon. Occasionally, a big he-goat would bound into their sanctum, with a loud ba-a; and above their heads were generally perched a whole brood of roosters, of all ages and all voices; some with colds and sore throats, but all eager to display their vocal accomplishments, in one unremitting crow. From midnight to daybreak, the challenge often flew from roost to roost, and every few minutes a full chorus was struck up. The smell, heat, and noises of the animals were intolerable, and the vermin active; yet, from three to five generations of human beings always live, cook, eat, and sleep in these foul holes. They are made by digging a tank in the ground, lined with rough stones for walls, covered overhead with huge timbers, and these again with sticks, bushes, and dry grass; then terraced over with a thick bed of earth, upon which sods and grass are planted.

---

Medical College of Alabama.—The friends of this college will be glad to learn that it has been reöpened. We quote from the circular received: "The Faculty has been reörganized and a full corps of professors are ready to devote their time and energies to the cause of medical education in the South." "The professors are all Southern men, born and nurtured on the soil of the South, and now, in the hour of her adversity, they are willing and anxious to do all in their power to raise her to that high destiny which no reverse of fortune or of war can snatch from her sorely tried and faithful sons." Terms: $140 for the session; demonstrator's ticket, $10; matriculation, $5; graduation, $30. Handsome premiums are offered for excellence in various branches. The circular is closed with an appeal to all, for encouragement and support.

